# A Review: Integrative Perspectives on the Features and Clinical Management of Psychotic Episodes in Pregnancy

**DOI:** 10.3390/jcm12020656

**Published:** 2023-01-13

**Authors:** Miguel A. Ortega, Tatiana Pekarek, Oscar Fraile-Martinez, Cielo García-Montero, Leonel Pekarek, Sonia Rodriguez-Martín, Rosa M. Funes Moñux, Coral Bravo, Juan A. De León-Luis, Guillermo Lahera, Jorge Monserrat, Javier Quintero, Julia Bujan, Natalio García-Honduvilla, Melchor Álvarez-Mon, Miguel Angel Alvarez-Mon

**Affiliations:** 1 Department of Medicine and Medical Specialities, Faculty of Medicine and Health Sciences, University of Alcalá, 28801 Alcalá de Henares, Spain; 2 Ramón y Cajal Institute of Sanitary Research (IRYCIS), 28034 Madrid, Spain; 3 Service of Pediatric, Hospital Universitario Principe de Asturias, 28801 Alcalá de Henares, Spain; 4 Department of Public and Maternal and Child Health, School of Medicine, Complutense University of Madrid, 28040 Madrid, Spain; 5 Department of Obstetrics and Gynecology, University Hospital Gregorio Marañón, 28009 Madrid, Spain; 6 Health Research Institute Gregorio Marañón, 28009 Madrid, Spain; 7 Psychiatry Service, Center for Biomedical Research in the Mental Health Network, University Hospital Príncipe de Asturias, 28806 Alcalá de Henares, Spain; 8 Department of Psychiatry and Mental Health, Hospital Universitario Infanta Leonor, 28031 Madrid, Spain; 9 Department of Legal Medicine and Psychiatry, Complutense University, 28040 Madrid, Spain; 10 Immune System Diseases-Rheumatology and Internal Medicine Service, University Hospital Príncipe de Asturias, CIBEREHD, 28806 Alcalá de Henares, Spain

**Keywords:** psychosis, pregnancy, antepartum psychosis, puerperal psychosis, clinical management

## Abstract

Psychotic episodes represent one of the most complex manifestations of various mental illnesses, and these encompass a wide variety of clinical manifestations that together lead to high morbidity in the general population. Various mental illnesses are associated with psychotic episodes; in addition, although their incidence and prevalence rates have been widely described in the general population, their correct identification and treatment is a challenge for health professionals in relation to pregnancy. In pregnant women, psychotic episodes can be the consequence of the manifestation of a previous psychiatric illness or may begin during the pregnancy itself, placing not only the mother, but also the fetus at risk during the psychotic episode. In addition, we cannot forget that both pharmacological and nonpharmacological management are complex given the different teratogenic effects of various neuroleptic drugs or mood stabilizers; moreover, the recommendation is that patients should be followed together with different specialists to maintain close contact during puerperium given the high incidence of recurrence of psychotic episodes. In addition, we cannot forget that a large portion of these patients for whom the onset times of such episodes are during pregnancy have a greater probability of an unpredictable psychiatric illness that requires a postpartum follow up, in addition to the postpartum psychotic episodes, at some point in their lives. Therefore, the purpose of this review is to summarize the epidemiology of psychotic breaks during pregnancy related to the main mental illnesses that affect this population and to summarize the main pharmacological treatments available for their clinical management.

## 1. Introduction and Psychosis during Pregnancy

Psychosis refers to a disorder in which the person suffers a loss of perception with respect to reality, where he or she cannot distinguish what is real from what is unreal. Psychosis has a wide spectrum of manifestations of different psychiatric illnesses, including delusions and visual, tactile or auditory hallucinations. The current DSM-V classification includes a section where entities that cover the broad spectrum of schizophrenia are grouped together with other psychotic disorders (e.g., short psychotic disorder lasting less than 1 month, psychosis induced by toxic substances), but without clarifying all the possible diagnostic possibilities [1]. One of the most relevant systematic reviews in regard to demonstrating the epidemiology of psychosis worldwide is based on the meta-analysis by Moreno-Kustner et al. [2], who evaluated 73 different articles and established that the global prevalence of psychotic episodes or psychosis in the population overall is 4.6 per 1000 people. In Spain, the data available by the Ministry of Health indicate that affective psychosis or bipolar disorder (which includes mania, hypomania, manic depression and cyclothymia) has a prevalence of 7.2 cases per 1000 inhabitants, being more frequent in women than in men (9.6‰ in women, 4.8‰ in men). On the other hand, in the broad spectrum of schizophrenia, it affects approximately 4.5 men out of every 1000 and 2.9 women out of every 1000, including disorders such as schizophrenia and schizotypal, delusional and schizoaffective disorders. In turn, there are two groups of psychoses, namely, unspecified psychoses, which range from acute psychotic episodes to reactive or puerperal psychoses and affect 1.9 out of 1000 people; and organic psychoses or acute confusional syndrome, which are much more difficult to quantify and whose prevalence rises beginning at 65 years, generally affecting 30 per 1000 people. The latter may be underdiagnosed since the confusional syndrome is usually the consequence of an organic disease and, in many cases, it is not considered [3].

The perinatal period encompasses both pregnancy and the subsequent 12 months. Throughout pregnancy, women may suffer from different psychiatric disorders, such as major depression, schizophrenia, bipolar disorder or maternal blues, postpartum depression and anxiety (which includes social phobia), as well as eating disorders, obsessive-compulsive disorder, posttraumatic stress disorder, panic attacks and puerperal psychosis [4]. During this period, there are a great variety of psychiatric emergencies that have to be considered given the repercussions that they can have on the fetus and the mother. Even so, we must mention that the prevalence of psychosis is very low during pregnancy, although its implications in the fetus are important. For instance, it has been reported that women who suffered a psychotic episode in pregnancy had an increased risk of multiple adverse obstetric and neonatal outcomes, such as cesarean delivery, poor fetal growth, placental abruption, antepartum/postpartum hemorrhage, fetal distress and abnormalities or stillbirth [5]. Among the main risk factors for suffering psychosis in pregnancy, having a family history of psychosis, previous history of psychosis in pregnancy and pre-existing or undiagnosed psychotic/mood disorder, such as schizophrenia or bipolar disorder, are considered [6,7]. The pathophysiological basis of psychosis in pregnancy is not completely understood. Changes in certain neurotransmitters, such as dopamine, acetylcholine, gamma aminobutyric acid (GABA) or glutamate, appear to be the main identified mechanisms in the brain [8]. However, these alterations seems not be the cause per se, but a response to a genetic and environmental background, which ultimately leads to neurotransmitter dysregulation in specific regions of the brain [9].

In reference to puerperal psychosis as a manifestation of previous illnesses, the incidence varies according to different authors because it is a delicate and complex subject to address and investigate and because numerous ethical-moral aspects must be considered when carrying out a clinical study. Even so, different authors estimate a prevalence of 7.1 out of 100,000 cases per year and others between 0.89 and 2.6 out of 1000 women [10]. All this is because although pregnancy itself is a protective factor against the development of psychotic disorders, the method and drugs used to treat them must be considered, since there are acute conditions during pregnancy, such as infections or diabetes, that can exacerbate the risk of suffering a psychotic episode [11]. For example, the risk of recurrence of manic episodes in patients with bipolar disorder during pregnancy is 52%; in addition to being the patients with the highest risk of suicide after childbirth, these patients have the highest risk of manic episodes during the postpartum period, with recurrence rates of up to approximately 70% [12,13,14]. Regarding schizophrenia, Fabre et al. and Simolia et al., who examined 3108 and 1162 patients with schizophrenia, respectively, have shown that these patients have higher rates of postpartum complications, such as gestational diabetes, preeclampsia, genitourinary infections, delayed intrauterine growth, cesarean sections or higher rates of abortion. They have also shown how these patients consume more tobacco, alcohol or other drugs than control patients [15,16].

Regarding the clinical manifestations of psychosis during pregnancy, the loss of perception of reality can be generated by thought disorders (delusions, nihilism), perception disorders (hallucinations) or even psychomotor disorders (catatonia, mannerisms, motor stereotypes) [17]. Normally, the psychotic episode lasts more than a day, but less than a month; in addition, after finishing, the person returns to his basal state and with it, to his normal functioning. Before these types of manifestations, other changes in behavior or social behavior may appear that are not so noticeable and/or extreme, but that may precede the onset of a disorder or outbreak in pregnant women. Completely different attitudes can be found in them, from extreme sociability to depressive attitudes, anguish, avoidance, lack of empathy or mistrust as manifestations of a manic picture in bipolar patients. There can also be changes in physical appearance caused by lack of personal care and hygiene produced by insomnia or alterations in diet, among others, in patients with schizophrenia [18].

Together, these observations indicate that it must be understood that women who suffer from previous disorders, such as schizophrenia, bipolar disorder or major depression, are more likely to develop a psychotic episode during this stage, to develop these diseases more quickly or even to suffer more episodes repeatedly after the pregnancy is over. We have carried out an exhaustive narrative review consulting all the existing scientific literature, as well as consensus guidelines and protocols; everything has been reflected in this document.

## 2. Treatment of Psychotic Episodes during Pregnancy

One of the most complex issues in the clinical management of a pregnant woman with a psychotic episode relies on the treatment of choice. There are multiple variables that must be considered, such as the prior diagnosis or new onset of a mental illness, medication previously used, trimester of pregnancy and risk of teratogenicity. We cannot forget that the information currently available to recommend a specific treatment in these cases is limited by the complexity of conducting randomized clinical trials due to the moral complexity of conducting pharmacological studies in pregnant patients. Despite this, one of the most relevant reviews to show the use of antipsychotics in pregnant women is the study by Toh et al. with 585,615 pregnant patients, of whom 4224 received treatment with second-generation antipsychotics and 548 received first-generation antipsychotics [19]. There are several relevant studies that address the teratogenic risks and perinatal complications associated with the use of antipsychotics. For example, one of the first meta-analyses carried out by Altshuler et al. [20] evaluated up to four studies that included 2591 pregnant women exposed to phenothiazines and showed that there was a slight increase in the risk of congenital anomalies in addition to postpartum complications such as respiratory depression or extrapyramidal symptoms when exposed to antipsychotic treatment in the first trimester; however, it should be noted that the included studies were difficult to evaluate. Along these lines, McKenna et al. [21] evaluated 151 pregnant women exposed to different types of antipsychotic drugs, such as olanzapine, risperidone and quetiapine; they found that the rate of congenital malformations was not higher than that of the control group of pregnant women not exposed to antipsychotics. In agreement with the previous results, Huybrechts et al. evaluated more than 1.3 million pregnant women, with 9258 having received antipsychotic treatment and 733 of the 9258 women receiving it during the first trimester; in addition, they studied the teratogenic repercussion after the use of typical antipsychotics versus atypical or non-use. Their results showed that there was no significant increase in the development of congenital problems in women medicated with these types of drugs compared to nonmedicated women, as far as the first trimester of pregnancy is concerned [22]. Regarding antipsychotic drugs, an increase in neonatal toxicity has been observed in the form of dyskinesias, agitation, sedation or respiratory distress due to the passage of different drugs through the placenta. The rate of placental passage from mother to fetus is important in drugs such as olanzapine (72%) or haloperidol (66%), in addition to other drugs such as risperidone (49%), which can limit the therapeutic arsenal in these patients [23].

**Figure 1:**
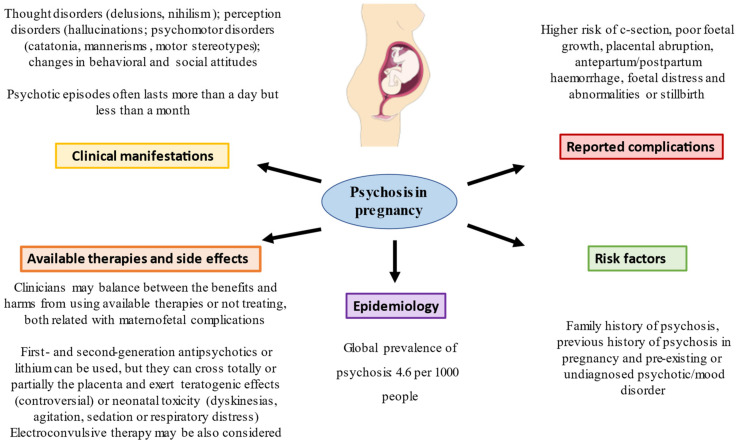
A summary of the main data of psychosis in pregnancy.

On the other hand, the standard treatment of patients with bipolar disorder is based on the use of lithium, which completely crosses the placenta, highlighting how the risk of teratogenicity can be up to double compared to that among healthy controls, mainly those with cardiac conditions, such as Ebstein’s malformation, alterations in the right ventricle, coarctation of the aorta and mitral atresia; in addition to the fact that at birth, the adverse effects of lithium generate apnea, bradycardia and seizures, among other adverse effects [24,25,26]. It should be mentioned that in these patients, the treatment of choice is based on the use of first-generation antipsychotics such as haloperidol, with the use of second-generation antipsychotics such as risperidone, quetiapine or olanzapine according to different authors [27,28]. In patients with bipolar disorder who do not respond to previous therapies, lithium could be used according to different guidelines, despite the possible adverse and teratogenic effects described [29]. Finally, we must mention that if all these measures are ineffective, there is the possibility of using electronconvulsive therapy, which is usually safe for both the mother and fetus and is effective, demonstrating partial remission rates in up to 70% of patients with bipolar disorder [30]. Therefore, given the complexity of the treatment in these patients, the general recommendations are usually based on evaluating the suspension of treatment if the underlying disease presents minimal symptoms or using the minimum possible dose to avoid polypharmacy, as well as informing the patient and relatives of the disease, the current situation and how to carry out more rigorous control. Despite this, treating or not treating these episodes and/or mental illnesses should be studied individually, where the benefit/risk ratio can be assessed. This is because it is difficult to be certain of the impact that these drugs may have during pregnancy since in clinical trials with human drugs, for ethical and legal reasons, the safety and well-being of people prevails before the development of science and society. Overall, epidemiological data, risk factors, clinical manifestations, complications and therapeutic approaches in psychosis in pregnancy are summarized in Figure 1.

## 3. Puerperal Psychosis

We must consider the much more well-known and frequent entity that is puerperal psychosis, which affects 1–2 women among 1000 deliveries. It usually appears in the first weeks of the postpartum period, being normally associated with patients with a history of bipolar disorder or with a greater risk of presenting the same in the future; however, approximately 50% of women do not have a history of previous psychiatric illnesses [31,32]. This entity is characterized by a confusional picture, the most prominent clinical manifestation of which is delirium and profound confusion, which can lead to nonorganic psychosis, such as mania, acute depression or schizophreniform disorder [33]. Major recognized risk factors include a history of postpartum psychosis with a 30% recurrence rate in subsequent deliveries, a history of mental illness with psychotic episodes or treatment modifications in patients with psychiatric illness during childbirth [34].

All this can have very negative consequences, especially in the mother-child relationship, where manifestations in the mother can be denial of the child; the theft or even the death of the child; or the belief that the child belongs to another mother, that they have changed the child or that they have changed the sex of the child. We must emphasize that this episode of psychosis has a sudden onset and generally occurs in the first two weeks after delivery, although we can find manifestations in the first days or even days before giving birth. Among them are sleep disorders with very pronounced insomnia, such as anxiety, asthenia or intense crying, where the woman becomes especially distant and distrustful of her surroundings [35,36]. If we add that the appearance of psychic symptoms such as hallucinations or delusions, a feeling of melancholy or, failing that, a persecutory state towards the child due to ideas of substitution, poisoning or denial of motherhood may appear; thus, endangering the life of the child or both the woman and the child due to suicide or infanticide. In a systematic review carried out by Gilden et al. that included 645 patients, evidence showed that the risk of suicide was up to 11% in patients with episodes of postpartum psychosis [37]. Currently, as we have previously mentioned, the DSM-V does not include this category as such; however, it includes it in the group of psychosis, indicating that the time of onset is peripartum. The differential diagnosis includes manic episodes in bipolar disorder, major depression with psychotic features, an acute psychotic episode during pregnancy and a substance-induced psychotic episode, in addition to acute confusional syndrome as a consequence of some underlying organic disease [38]. After the resolution of these episodes, the woman returns to her normal pre-psychotic state and, in approximately 80% of cases, there is a full recovery, although the high likelihood of relapse in the future must be considered [39]. Therefore, we can observe how there are two clearly differentiated entities that are the puerperal psychosis of the antepartum psychosis, each one with a special handling method and characteristics, with puerperal psychosis much more frequent than antepartum psychosis.

## 4. Puerperal Psychosis Treatment

**Figure 2:**
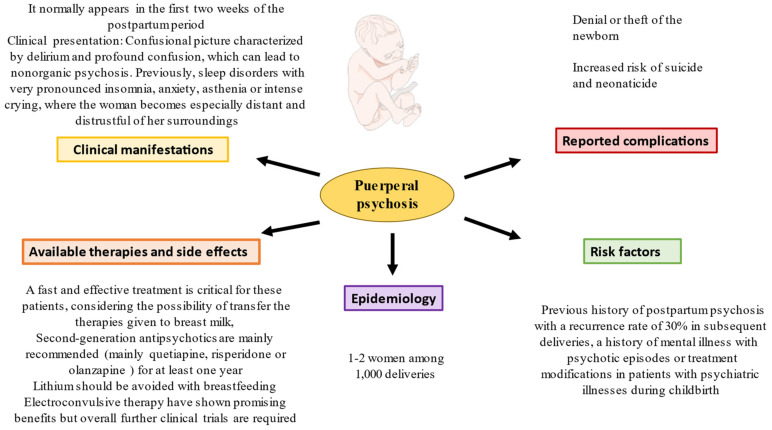
A summary of the main data of puerperal psychosis.

As with the treatment of psychotic episodes during pregnancy, in the puerperium period, many aspects must be considered, such as the transfer of different antipsychotics to breast milk and that the rapidity of the presentation of the picture of puerperal psychosis entails applying a fast and effective treatment, given the prognostic implications associated with the psychosis in terms of self-aggression and the hetero-aggressiveness of the patient towards the newborn. Therefore, it should be considered an emergency in psychiatry and promptly addressed. It should also be noted that the treatment of puerperal psychosis, as well as psychosis during childbirth, lacks randomized clinical trials to evaluate the effective ones among different options, cases or small subgroups of patients [40]. The first thing that must be considered is the safety of both the newborn and the mother; in addition, since the conditions are not present in most cases involving the safety of the newborn, the recommendation is to maintain vigilance in the interaction between the mother and newborn at hospital admission. Given the different possible manifestations of postpartum psychosis in cases in which agitation or psychosis predominates, the recommendation is to start therapy with second-generation antipsychotics, which are not passed at high quantities to breast milk and have lower rates of extrapyramidal effects and tardive dyskinesia than first-generation antipsychotics; quetiapine, risperidone or olanzapine is preferred, given the higher levels of clinical experience with these drugs since they are the ones that have been used the most in the different case reports [41,42,43]. Subsequently, the recommendation is to maintain treatment with antipsychotics for at least one year and subsequently, evaluate these patients [44]. In cases where patients have severe insomnia, the use of short-term benzodiazepines such as lorazepam in combination with second-generation antipsychotics is recommended [45]. On the other hand, we must remember that a significant percentage of these patients have bipolar disorder, which is why mood stabilizers have an important relevance in these patients; in particular, lithium, which cannot be administered if the child is going to breastfeed, and valproic acid can be used in this case [46,47]. Finally, as in antepartum psychosis in refractory cases, electroconvulsive therapy can be used, as it has good results in different case reports. For example, Reed et al. evaluated the use of electroconvulsive therapy in 58 women with puerperal psychosis and showed improvement in up to 65% of cases [48]. These results are in line with those observed by Rundgren et al., who observed a response rate of up to 87% in 185 women with postpartum psychosis [49]. Finally, we must remember that socio-family support is important for follow up, in addition to the fact that the patient should be followed up after the postpartum period as well as if there are wishes for additional pregnancies given the possibility of recurrence of this entity [50]. Collectively, epidemiological data, risk factors, clinical manifestations, complications and therapeutic approaches in puerperal psychosis are summarized in Figure 2.

## 5. Prevention

One of the most important aspects of mental health in pregnant women is based on early follow up of patients at risk. As with chronic diseases, such as diabetes, hypertension or other endocrinological disorders, mental health is a relevant aspect of obstetric care. The prevalence of psychiatric illness in the population is high; however, in women of reproductive age, the prevalence can be up to 70% as evidenced in the systematic review by Bezerra et al. [51], which includes 19 articles including 45,400 women from different countries [51]. In this line, different authors such as Lassi et al. [52] have shown in a systematic review that even though early interventions in endocrinological diseases are accompanied by a lower rate of complications during pregnancy in psychiatric diseases, there is no optimal preventive management [52]. It should be noted that different authors have shown that interventions through psychotherapy help to reduce the incidence of anxiety or other mental disorders during pregnancy [53,54]. In this line, authors such as Dimidjian et al. [55] have shown that there are different non-pharmacological therapies to prevent the onset of depression during pregnancy and postpartum, which demonstrates that, in most cases, it is not necessary to use drugs that may affect the fetus [55]. Therefore, we can observe that the rest of the comorbidities during childbirth present different prevention techniques, while mental health has been relegated to the background even though different non-pharmacological therapies have demonstrated their use and benefit for women’s mental health.

## 6. Conclusions

The prevalence of mental illnesses, such as major depression, bipolar disorder or schizophrenia, in the general population that can manifest as psychotic episodes is high enough to affect a large number of women during pregnancy. These psychotic episodes can be of new origin or an exacerbation of a previous illness and they can be associated with substance use. Two main presentations can be distinguished depending on the time of onset of psychosis: psychosis in pregnancy or puerperal/postpartum psychosis. A comparison between these two entities is given in Table 1. Clinicians face an extraordinarily complex challenge in these cases since both not acting and implementing available therapeutic regimens can have detrimental consequences for the fetus and mother. First- or second-generation antipsychotics are common therapeutic alternatives used as well as lithium or mood stabilizers for those women with previous psychiatric illnesses. However, given the teratogenic or perinatal implications of these pharmacotherapies, each patient should be evaluated individually with a multidisciplinary team, in addition to maintaining a postpartum follow up given the high possibility of recurrence. Electroconvulsive therapy has shown some promising, but still preliminary, evidence of its use; in addition, further trials are warranted in this area, also exploring potential translational approaches for these women. Therefore, the complexity of the management and follow up of these patients must be considered by every psychiatrist, gynecologist and primary care physician who is going to evaluate and maintain the continuous care of these patients.

## Figures and Tables

**Table 1:** Main characteristics of antepartum and puerperal (postpartum) psychosis.

	Antepartum Psychosis	Puerperal Psychosis	References
Incidence	0.89 out of 1000 pregnant women	1–2 women per 1000 deliveries	[10,11,31]
Associated psychiatric diseases	Mainly bipolar disorder	In all, 50% have no history of previous psychiatric illnesses.	[12,32]
Treatment	Second-generation antipsychotics, lithium or electroconvulsive therapy in refractory cases	Second-generation antipsychotics, lithium or electroconvulsive therapy in refractory cases	[27,28,29,30,41,42,43,44,45,46,47]
Prognosis	~52% recurrence in women with bipolar disorder	If they have presented puerperal psychosis, they have a ~30% risk of recurrence in other pregnancies. Suicide risk of ~11%	[13,14,34,37]

## Data Availability

Not applicable.
